# Activation of retinoid X receptor by bexarotene attenuates neuroinflammation via PPARγ/SIRT6/FoxO3a pathway after subarachnoid hemorrhage in rats

**DOI:** 10.1186/s12974-019-1432-5

**Published:** 2019-02-21

**Authors:** Yuchun Zuo, Lei Huang, Budbazar Enkhjargal, Weilin Xu, Ocak Umut, Zachary D. Travis, Guangyu Zhang, Jiping Tang, Fei Liu, John H. Zhang

**Affiliations:** 1 Department of Neurosurgery, Third XiangYa Hospital, Central South University, No.138 Tongzipo Road, Changsha, 410013 China; 2 Department of Physiology and Pharmacology, Loma Linda University, 11041 Campus St. Risley Hall, Loma Linda, CA 92354 USA; 3 Department of Neurosurgery, School of Medicine, Loma Linda University, Loma Linda, CA 92354 USA; 4 Department of Anesthesiology, School of Medicine, Loma Linda University, Loma Linda, CA 92354 USA; 5 Mass Spectrometry Core Facility, Loma Linda University, Loma Linda, CA 92354 USA; 6 Department of Earth and Biological Sciences, School of Medicine, Loma Linda University, Loma Linda, CA 92354 USA

**Keywords:** Subarachnoid hemorrhage, Retinoid X receptor, Bexarotene, Sirtuin 6, Neuroinflammation, Brain edema

## Abstract

**Background:**

Subarachnoid hemorrhage (SAH) is a life-threatening subtype of stroke with high mortality and disabilities. Retinoid X receptor (RXR) has been shown to be neuroprotective against ischemia/reperfusion injury. This study aimed to investigate the effects of the selective RXR agonist bexarotene on neuroinflammation in a rat model of SAH.

**Methods:**

Two hundred male Sprague-Dawley rats were used. The endovascular perforation induced SAH. Bexarotene was administered intraperitoneally at 1 h after SAH induction. To investigate the underlying mechanism, the selective RXR antagonist UVI3003 and RXR siRNA or SIRT6 inhibitor OSS128167 was administered via intracerebroventricular 1 h before SAH induction. Post-SAH assessments including SAH grade, neurological score, brain water content, Western blot, and immunofluorescence were performed.

**Results:**

The endogenous RXR and sirtuin 6 (SIRT6) protein levels were increased after SAH. Bexarotene treatment significantly reduced brain edema and improved the short-/long-term neurological deficit after SAH. Mechanistically, bexarotene increased the levels of PPARγ and SIRT6; decreased the expression of phosphorylated FoxO3a (p-FoxO3a), IL-6, IL-1β, and TNF-a; and inhibited the microglia activation and neutrophils infiltration at 24 h after SAH. Either UVI3003, OSS128167, or RXR siRNA abolished the neuroprotective effects of bexarotene and its regulation on protein levels of PPARγ/SIRT6/p-FoxO3a after SAH.

**Conclusions:**

The activation of RXR by bexarotene attenuated neuroinflammation and improved neurological deficits after SAH. The anti-neuroinflammatory effect was at least partially through regulating PPARγ/SIRT6/FoxO3a pathway. Bexarotene may be a promising therapeutic strategy in the management of SAH patients.

**Electronic supplementary material:**

The online version of this article (10.1186/s12974-019-1432-5) contains supplementary material, which is available to authorized users.

## Introduction

Subarachnoid hemorrhage (SAH) is a devastating and life-threatening cerebrovascular disease with high mortality and disability. It accounts for 5% of all stroke subtypes, whereas caused 50% case fatality after aneurysm disruption [1]. Despite the availability of early imaging diagnosis and neurosurgical interventions (clipping) as well as platinum spirals (coiling), the high mortality and poor outcome of SAH result in a high socioeconomic burden [2]. Neuroinflammation is considered as one of the main pathological processes in the early brain injury (EBI) after SAH [3]. The brains residual microglial/astrocytes activation and peripheral immune cells infiltration release massive pro-inflammatory cytokines which would magnify the inflammatory responses and further aggravate the neurological deficit [4]. Herein, the therapeutic strategies targeting neuroinflammation would be practical to minimize EBI and improve neurologic outcomes after SAH.

Retinoid X receptor (RXR) is a member of the NR2B nuclear receptor family. As a common binding partner of many other nuclear receptors, it mainly functions as a ligand-dependent transcription factor in regulating a plethora of physiological processes [5, 6]. RXR has been shown to bind with peroxisome proliferator-activated receptor gamma (PPARγ) to form heterodimers [7]. PPARγ belongs to the nuclear hormone receptor superfamily and exerts an anti-inflammatory effect in many neurological diseases [8–12]. RXR agonists can activate RXR/PPARγ heterodimers to inhibit inflammatory response [7]. Recent studies demonstrated that PPARγ activation induced the expression of sirtuin 6 (SIRT6) in a feed-forward manner [13, 14]. Also, SIRT6-mediated suppression of forkhead box O3a (FoxO3a) phosphorylation protected cardiomyocytes against ischemia/reperfusion injury [15]. Thus, the regulation of the RXR/PPARγ/SIRT6/FoxO3a pathway may serve as a potential anti-neuroinflammation strategy in the setting of SAH.

Bexarotene (PubChem CID: 82146), a highly selective and blood-brain barrier permeable RXR agonist, is a member of the retinoic acid family [16]. It has been initially approved by the U.S. Food and Drug Administration (FDA) for treating cutaneous lymphoma with a favorable safety profile [17]. Recent studies suggested that bexarotene was neuroprotective against a variety of neurological diseases, such as Alzheimer’s disease, traumatic brain injury, and ischemic stroke [16, 18, 19]. However, the neuroprotective effect of bexarotene and the underlying mechanisms have not been investigated in SAH.

In the present study, we hypothesized that bexarotene could attenuate neuroinflammation and improve neurological outcomes in a rat model of SAH. Such neuroprotective effects were mediated through PPARγ/SIRT6/FoxO3a signaling pathway.

## Materials and methods

### Animals and the SAH models

Adult male Sprague-Dawley rats (*n* = 200; 280–320 g) were housed in a room with constant temperature (25 °C) and humidity control, under a 12-h light/dark cycle and free access to food and water. All the experimental procedures were approved by the Institutional Animal Care and Use Committee at Loma Linda University and in accordance with the National Institutes of Health Guide for the Care and Use of Laboratory Animals of the National Institutes of Health.

Experimental SAH model was induced in rats using a modified endovascular perforation method as previously described [20, 21]. Briefly, rats were anesthetized by 5% isoflurane. After intubation, the rats were ventilated with 3% isoflurane in mixed gases (65% medical air with 35% oxygen gas) during surgery. Rats were operated in the supine position, and the midline incision was made in the neck. After carefully exposing the left carotid artery, a 3-cm-long sharpened monofilament nylon suture (4–0) was inserted into the external carotid artery, advanced via the internal carotid to the bifurcation of the anterior and middle cerebral arteries where the resistance was felt, and then further advanced slightly (3 mm) to induce a hemorrhage. Afterward, the nylon suture was withdrawn immediately. The sham-operated animals underwent the same surgical procedure without arterial perforation. At the end of surgery, the incision was closed, and the tracheal tube was extubated. The rats were housed individually in heated cages after recovery from anesthesia.

### Experimental design

**Fig. 1:**
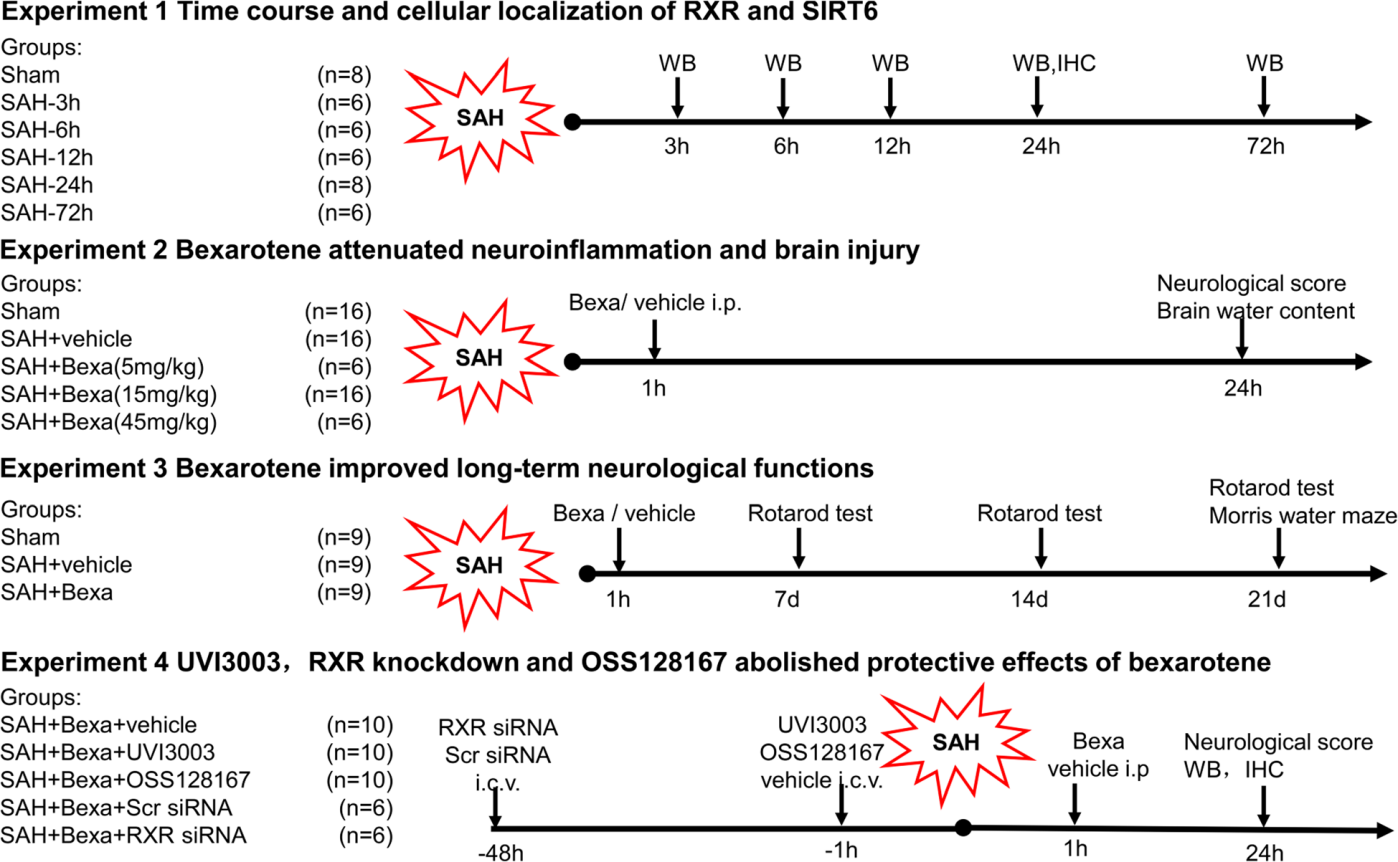
Experimental design and animal groups. *RXR* retinoid X receptor, *SIRT6* sirtuin 6, *Bexa* bexarotene, *UVI3003* RXR antagonist, *OSS128167* selective sirtuin 6 inhibitor, *IHC* immunohistochemistry, *WB* western blot, *i*.*p* intraperitoneal injection, *i*.*c*.*v* intracerebroventricular injection, *SAH* subarachnoid hemorrhage

Animals were randomly assigned to four separate experiments as following described. The information of experimental groups was blinded to the researchers who performed the surgeries, neurobehavioral assessments, Western blot, immunofluorescence staining, and data analysis. The experimental designs are schematically shown in Fig. 1.

#### Experiment 1

The time course and cellular localization of RXR and SIRT6 in the left hemisphere were accessed by Western blot and double immunofluorescence. Total of 36 rats were divided randomly and assigned into 6 groups with *n* = 6/group: sham and SAH 3, 6, 12, 24, and 72 h (*n* = 6/group). The temporal pattern of RXR and SIRT6 expression was detected by Western blot. The additional four rats in sham and SAH 24 h group (*n* = 2/group) were used for double immunofluorescence staining to show the co-localization of RXR with the neurons, astrocytes, and microglia.

#### Experiment 2

To assess the neuroprotective effects of bexarotene (Bexa), a total of 30 rats were randomized into 5 groups with *n* = 6/group: sham, SAH + vehicle, SAH + Bexa (5 mg/kg), SAH + Bexa (15 mg/kg), and SAH + Bexa (45 mg/kg). Bexarotene was administered at 1 h after SAH by intraperitoneal (i.p) injection. Neurological functions and brain water content were measured at 24 h after SAH. Based on neurological tests and brain water content results, bexarotene at a dose of 15 mg/kg was the most effective. Thus this best dosage was chosen for the following long-term neurobehavior and mechanism experiments.

To explore the effects of bexarotene on microglia/macrophage activation and neutrophil infiltration at 24 h after SAH, another set of 30 rats were randomly divided into 3 groups with *n* = 10/group: sham, SAH + vehicle, and SAH + Bexa (15 mg/kg). Western blot (*n* = 6/group) and immunofluorescence staining (*n* = 4/group) were performed to detect the ionized calcium binding adaptor molecule 1 (Iba-1)-positive microglia and myeloperoxidase (MPO)-positive neutrophils at 24 h after SAH. Brain samples of these three groups were shared with experiment 4.

#### Experiment 3

To evaluate the effects of bexarotene on long-term neurobehavioral recovery, 27 rats were randomly divided into 3 groups with *n* = 9/group: sham, SAH + vehicle, and SAH + Bexa (15 mg/kg). The rotarod tests were performed on days 7, 14, and 21 after SAH and Morris water maze test were performed on days 21–25 after SAH.

#### Experiment 4

To explore the potential mechanism of RXR/PPARγ/SIRT6/FoxO3a underlying Bexa mediated anti-neuroinflammation effects after SAH, the selective RXR inhibitor UVI3003 or selective SIRT6 antagonist OSS128167 was administration by intracerebroventricular (i.c.v) injection 1 h before SAH. To further prove the pathway, the RXR siRNA was administration by i.c.v injection 48 h before SAH. In addition to the shared brain samples of sham, SAH + vehicle, SAH + Bexa groups from experiment 2, additional 42 rats were assigned into 5 groups: SAH + Bexa + vehicle (*n* = 10/group), SAH + Bexa + UVI3003 (*n* = 10/group), SAH + Bexa + OSS128167 (*n* = 10/group), SAH + Bexa + Sramble siRNA (*n* = 6/group), and SAH + Bexa + RXR siRNA (*n* = 6/group). Neurological tests and Western blot and immunofluorescence were performed at 24 h after SAH.

### Drug administration

Bexarotene (Selleck Chemicals, Houston, USA) was diluted in 10% dimethyl sulfoxide (DMSO). Administered 1 h after SAH by i.p injection, three different doses (5 mg/kg, 15 mg/kg, and 45 mg/kg) were evaluated. The SAH + vehicle group received an equal volume of 10% DMSO. The RXR inhibitor UVI3003 (75 μg/rat, Sigma-Aldrich, St. Louis, MO) or selective SIRT6 antagonist OSS128167 (100 μg/rat, Selleck Chemicals, Houston, TX) dissolved in 10% DMSO was injected by i.c.v route at 1 h before SAH. The same volume of 10% DMSO was injected via i.c.v in SAH + Bexa + vehicle group.

### Intracerebroventricular injection

An intracerebroventricular injection was performed as described previously [22, 23]. Briefly, rats were placed in a stereotaxic apparatus under anesthesia with 3% isoflurane in mixed gases (70% medical air/30% oxygen). A cranial burr hole was drilled at the following coordinates relative to bregma: 1.5 mm posterior, 1.0 mm lateral. The 10-μL Hamilton syringe (Microliter701; Hamilton Company, Reno, NV, USA) was inserted at a depth of 3.3 mm into the right lateral ventricle through the burr hole as mentioned above. The needle was inserted through the burr hole. The rate of injection controlled at 1 μL/min with an infusion pump (Stoelting, Harvard Apparatus, Holliston, MA, USA). For RXR knockdown in vivo, rat RXR siRNA (Santa Cruz Technology, USA) or Scramble siRNA was prepared in RNase free suspension buffer at a concentration of 100 pmol/uL and then infused into the right lateral ventricle at 48 h before SAH induction. The needle was kept in place for an additional 5 min after the end of infusion and then slowly retracted throughout 5 min. The burr hole was sealed with bone wax, and the rats were allowed to recover after sutures.

### SAH grading score

The severity of SAH was evaluated according to a grading system as previously described [24]. Briefly, the basal cistern was divided into six segments and scoring based on the number of subarachnoid blood clots respectively by the following criteria: 0, no subarachnoid blood; 1, minimal subarachnoid clots; 2, moderate subarachnoid clots with recognizable arteries; and 3, blood clots covering all arteries. The total score was obtained by adding the scores of all six segments. Rats with mild SAH (SAH grade score less than 8) at 24 h were excluded from this study. The grading was performed by researchers blinded to the experiment group information.

### Assessment of short-term neurological function

Short-term neurological function was assessed using the modified Garcia scoring system and the beam balance test at 24 h after SAH, as previously described [25]. The Garcia score was composed of six test subscores, including spontaneous activity, spontaneous movement of the four limbs, body proprioception, whisker proprioception, forepaw outstretching, and climbing. The total score ranging from 3 to 18 was obtained by adding the six test subscores [26]. Beam balance tests evaluated the ability of animals to walk on a narrow cylindrical wooden beam for 60 s. The score was evaluated as follows: 0, no walking and falling; 1, no walking, but remains on the beam; 2, walking but falling; 3, walking less than 20 cm; 4, walking beyond 20 cm. These neurological tests with high scores indicated better neurological function.

### Assessment of long-term neurological function

Long-term neurobehavioral effects were assessed using the rotarod test and Morris water maze test as previously reported [27]. The rotarod test was performed to measure the balance and coordination abilities. Briefly, the rats were placed on the rotarod at the starting rate of 5 rpm and 10 rpm with an acceleration of 2 revolutions per 5 s. The duration of rats on the rotarod were recorded and used for statistical analysis. The Morris water maze was used to evaluate the abilities of spatial learning and memory on days 21–25 after SAH. Briefly, the rats were placed in different start locations using a semi-random method to find a visible platform above the water level in 60 s. On the last day, probe trial test were performed with the platform removed. Swim trace and distance, escape latency, and probe quadrant duration were recorded by a computerized tracking system (Noldus Ethovision; Noldus, Tacoma, WA, USA).

### Brain water content

Brain water content was evaluated by a wet/dry method, as described previously [28]. The brains were separated into four parts (left hemisphere, right hemisphere, cerebellum, and brain stem), and weighed immediately (wet weight), then weighed again after dried at 105 °C for 72 h (dry weight). The percentage of brain water content was calculated by [(wet weight − dry weight)/wet weight] × 100%.

### Liquid chromatography-mass spectrometry

The liquid chromatography-mass spectrometry (LC-MS/MS) detection was performed as previously described [29]. The brain samples were prepared to follow the reported method [30]. Briefly, 200 mg of brain tissue was homogenized in 2 mL of acetonitrile (Sigma-Aldrich, USA), and then centrifuged at 14,000 g for 30 min at 4 °C. The supernatant was collected and dried under negative pressure (below 2.0 kPa) for 7 h at 4 °C. The residue was reconstituted with 1000 μL 50% acetonitrile and centrifuged at 14,000×*g* for 10 min at 4 °C. Then, 20 μL of the supernatant was injected into the LC-MS/MS system. The MS spectra were collected under the positive reflector mode from *m*/*z* 100–1000. MS/MS spectra were acquired using collision energy of 30 kV with the metastable suppressor on. The LC-MS/MS data was visualized and analyzed by MassHunter Software Version B.08.00 (Agilent Technologies, CA, USA).

### Western blot analysis

Western blotting was performed as described previously [31]. Briefly, the samples were extracted in RIPA buffer (Santa Cruz Biotechnology, CA, USA) and centrifuged with 14,000×*g* at 4 °C for 30 min. The supernatant was collected and followed by a protein concentration measurement using detergent compatible assay (DC protein assay, Bio-Rad Laboratories, CA, USA). Equal amounts of protein samples were separated by SDS-PAGE gel and transferred to nitrocellulose membrane. Afterward, the membranes were blocked and incubated overnight at 4 °C with the following primary antibodies: anti-RXR (1:300; Santa Cruz Biotechnology, Santa Cruz, CA, USA), anti-PPARγ (1:1000; Abcam, Cambridge, MA, USA), anti-SIRT6 (1:1000; Abcam, Cambridge, MA, USA), anti-phosphorylated FoxO3a (p-FoxO3a, 1:1000 Abcam, Cambridge, MA, USA), anti-FoxO3a (1:1000, Cell Signaling, Danvers, MA, USA), anti-Iba-1 (1:1000, Abcam, Cambridge, MA, USA), anti-MPO (1:500, Abcam, Cambridge, MA, USA), anti-IL-1β (1:1000, Abcam, Cambridge, MA, USA), anti-IL-6 (1:1000, Abcam, Cambridge, MA, USA), anti-TNF-α (1:1000, Abcam, Cambridge, MA, USA), and anti-β-actin (1:3000, Santa Cruz, Dallas, TX, USA). Appropriate secondary antibodies (1:3000, Santa Cruz, Dallas, TX, USA) were incubated at room temperature for 2 h. The specific bands were visualized by an ECL reagent (Amersham Biosciences, Pittsburgh, PA). The relative densities of the immunoblot bands were analyzed using ImageJ software (Image J 1.4, NIH, USA).

### Immunoprecipitation (IP) detection

The left hemisphere was lysed and extraction followed by centrifugation. The primary antibodies (5 μg, anti-RXR, Santa Cruz Biotechnology, USA) were pre-incubated at room temperature mixed with 30 μL agarose-G for 5 h. After rinsed with GLB + buffer for five times, the brain lysate was added and incubated with agarose-G combined with primary antibody at 4 °C for 24 h. After that, the mixed proteins were washed with pre-cold GLB + buffer three times, followed by eluted with 1 × loading buffer in boiling water for 8 min, and collected the supernatant after centrifuged at 12,000 rpm for 2 min. The supernatant was loaded to SDS-PAGE.

### Immunofluorescence

Rats were under deep anesthesia and transcardially perfused with pre-cold PBS and 10% formalin. The brains were post-fixed in 10% formalin at 4 °C for 24 h followed by dehydrated in 30% sucrose for another 72 h. Brain samples were frozen at − 80 °C after embedding in OCT and cut into 10-μm-thick coronal sections using a cryostat (CM3050S; Leica Microsystems, Bannockburn, III, Germany). To perform the immunofluorescence staining, the slices were rinsed and blocked with 5% donkey serum at room temperature for 1 h, then incubated overnight at 4 °C with the following primary antibodies: goat anti-Iba-1 (1:200, Abcam, Cambridge, MA, USA), goat anti-GFAP (1:200, Abcam, Cambridge, MA, USA), goat anti-NeuN (1:200, Abcam, Cambridge, MA, USA), mouse anti-RXR(1:100; Santa Cruz, Dallas, TX, USA), rabbit anti-SIRT6 (1:200, Abcam, Cambridge, MA, USA), rabbit anti-IL-1β (1:100, Abcam, Cambridge, MA, USA), and mouse anti-MPO (1:100, Santa Cruz, Dallas, TX, USA). After that, slides were incubated with the corresponding secondary antibodies (1:200, Jackson Immunoresearch, West Grove, PA, USA). The sections were visualized and photographed under a fluorescence microscope (Leica Microsystems, Germany). Microphotographs were analyzed with LASX software. The numbers of Iba-1-positive cells, MPO-positive cells, and IL-1β positive cells were identified and counted in three different fields of the left basal cortex from five random coronal sections per rat, and data were expressed as cells/field.

### Quantification and statistical analysis

The sample size was calculated based on the literature reviewing and our previous experimental SAH studies as well as the preliminary data collected for the treatment efficacy of Bexarotene against stroke. Using sample size calculator (SigmaPlot) with a power of 0.8, and an alpha of 0.05 on a two-sided test, the animal numbers *n* = 6/group was deemed sufficient to the experiments. All data were expressed as mean and standard deviation (means ± SD) and plotted using Graph Pad Prism 7 (Graph Pad Software, San Diego, CA). SPSS 16.0 software (SPSS Inc., Chicago, Illinois, USA) was used for statistical analysis. One-way analysis of variance (ANOVA) followed by Tukey’s post-hoc test was used for multiple comparisons among groups. Two-way ANOVA was applied to analyze long-term neurobehavioral results. *P* < 0.05 was defined as statistical significance.

## Results

### Animal mortality and SAH severity

A total of 200 rats were used, in which 33 rats underwent a sham operation, and 163 rats underwent SAH induction. Out of 163 SAH rats, 4 rats were excluded because their SAH grading was less than 8. The total mortality of SAH rats in the study was 16.56% (27/163). None of the rats died in the sham-operated group. The SAH mortality rate was not significantly different among the experimental groups. The average SAH grades among all the SAH groups were not statistically different. Blood clots were mainly distributed around the Circle of Willis and ventral brain stem after SAH induction, whereas no blood clot was observed in the sham operation (Additional file 1: Figure S1).

### Temporal patterns and cellular expression of endogenous brain RXR and SIRT6 after SAH

**Fig. 2:**
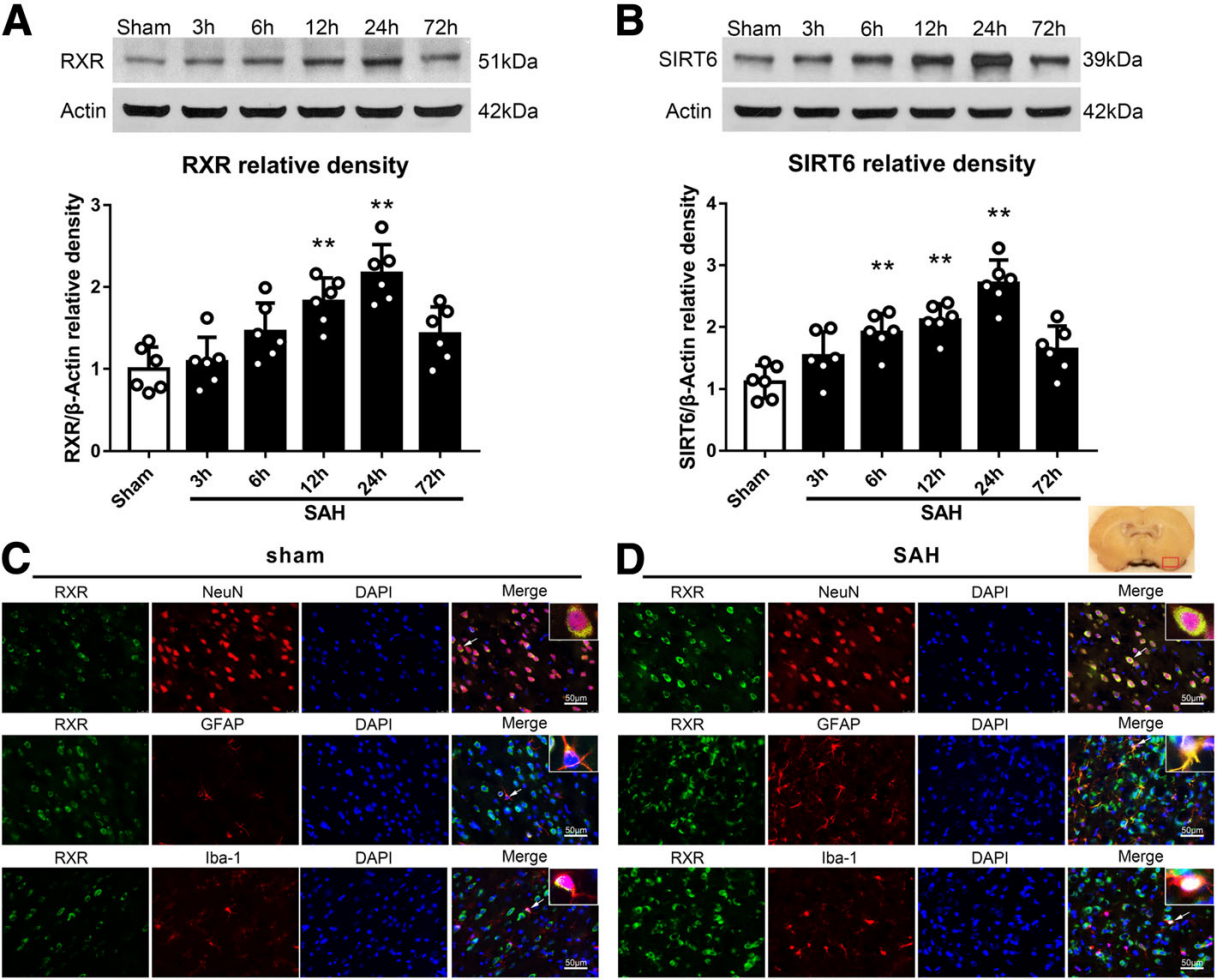
Temporal expressions and cellular localization of endogenous brain RXR and SIRT6 after SAH. **a** Representative western blot bands and quantitative analyses of RXR time course in the left hemisphere after SAH. **b** Representative western blot bands and quantitative analyses of SIRT6 time course in the left hemisphere after SAH. *P < 0.05 vs sham group. Error bars were represented as mean ± SD. *n* = 6 per group. **c** Double immunofluorescence staining for RXR (green) in the neuron (NeuN, red), astrocytes (GFAP, red), and microglia (Iba-1, red) in the left basal cortex at 24 h after SAH. **d** Double immunofluorescence staining for SIRT6 (red) in the neuron (NeuN, green), astrocytes (GFAP, green), and microglia (Iba-1, green) in the left basal cortex at 24 h after SAH. *n* = 2 per group. Scale bar, 50 μm. DAPI indicates 4′,6-diamidino2-phenylindole; *GFAP* glial fibrillary acidic protein, *Iba*-*1* ionized calcium binding adaptor molecule-1, *NeuN* neuronal nuclear

Western blotting was performed to detect the protein levels of RXR and SIRT6 in the left hemisphere among groups of sham, 3, 6, 12, 24, and 72 h after SAH. The results showed that the expression of RXR and SIRT6 started increasing at 3 h after SAH and peaked at 24 h (*P* < 0.05; Fig. 1a, b). The RXR and SIRT6 protein levels at 24 h post-SAH were nearly three times higher than those in the sham group. Double immunofluorescence staining of the RXR with the neurons marker NeuN, astrocytes marker glial fibrillary acidic protein (GFAP), or microglia marker Iba-1 showed that RXR expressed on the three types of cells within cortices in sham and SAH rats at 24 h after injury (Fig. 2c, d).

### Bexarotene treatment improved short-term neurobehavioral deficits and reduced brain edema at 24 h after SAH

**Fig. 3:**
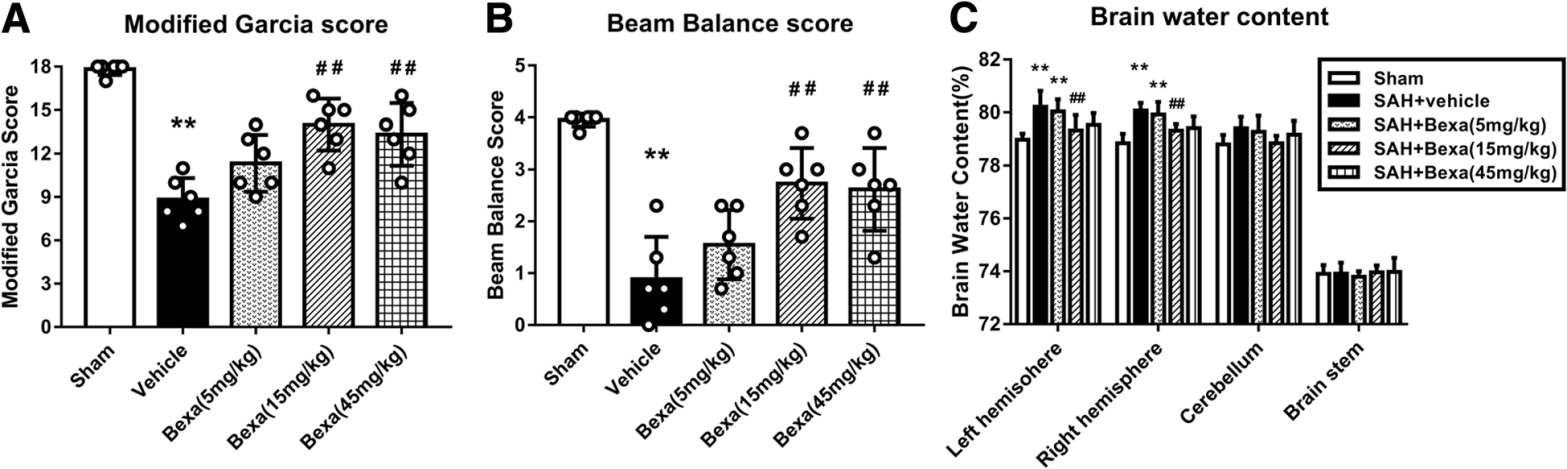
Bexarotene attenuated the neurobehavioral deficits and brain edema at 24 h after SAH. **a** Modified Garcia score and beam balance score. **b** Brain water content in the left hemisphere, right hemisphere, cerebellum, and brain stem. Error bars were represented as mean ± SD. *n* = 6 per group. *Bexa* bexarotene

The neurobehavioral outcomes and brain water content were evaluated at 24 h after SAH. Rats in the SAH + vehicle groups performed significantly worse than shams in the modified Garcia test and beam balance test (*P* < 0.01; Fig. 3a, b). The administration of bexarotene (15 mg/kg and 45 mg/kg) significantly improved the neurological scores at 24 h after SAH. The brain water content in the left and right hemisphere was significantly increased in the SAH groups compared to sham at 24 after SAH (*P* < 0.05, Fig. 3c), which was significantly reduced by the administration of Bexa at a dose of 15 mg/kg (*P* < 0.05, Fig. 3c). The brain water contents in the cerebellum and brain stem were not significant differences between the sham and SAH groups. Bexarotene at 15 mg/kg was the most effective dosage, which was chosen for the following long-term and mechanistic studies.

### Bexarotene treatment reduced microglia/macrophage activations and neutrophil infiltration at 24 h after SAH

**Fig. 4:**
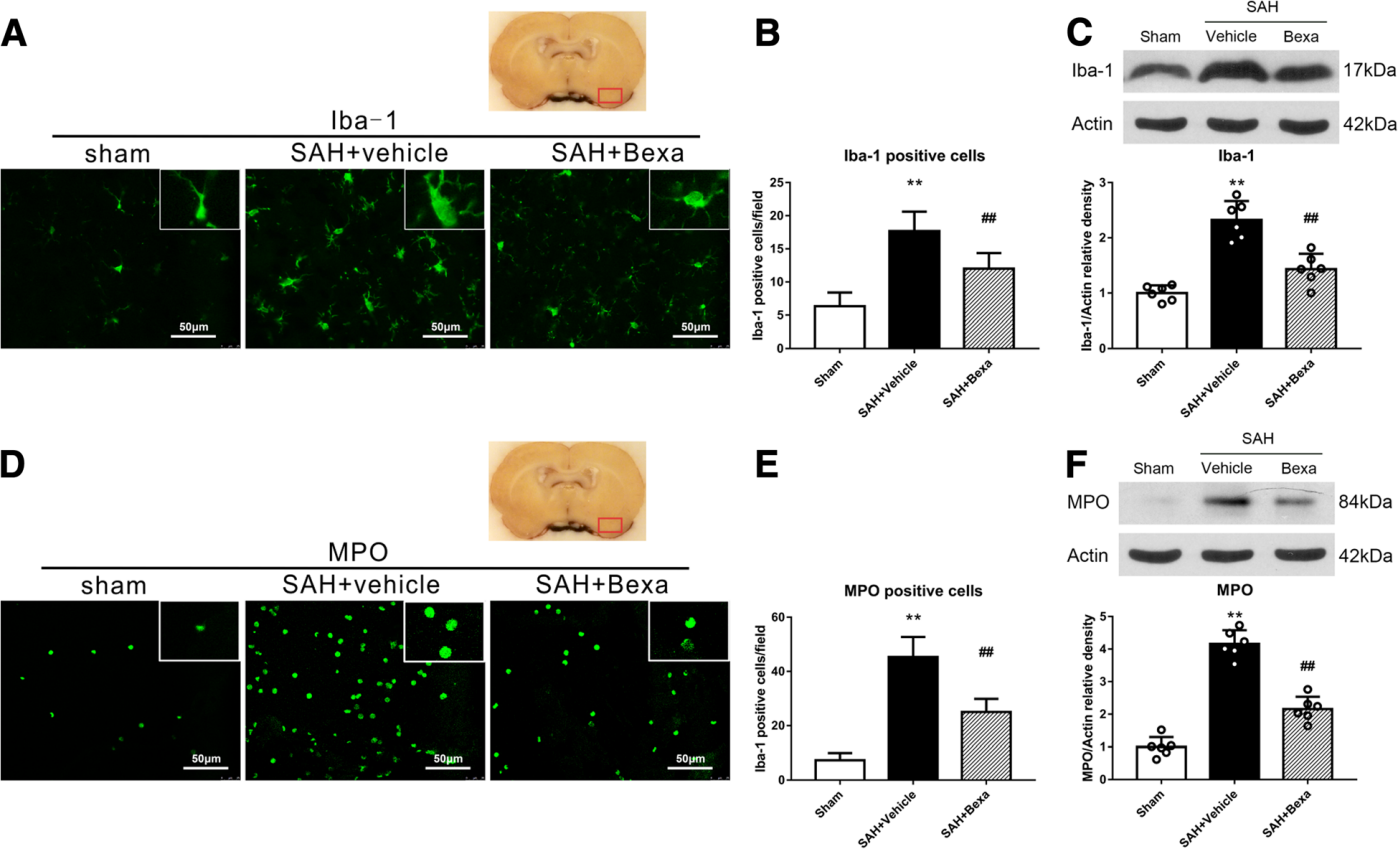
The effects of bexarotene on microglia/macrophage activation and neutrophil infiltration at 24 h after SAH. **a**, **d** Representative microphotograph of immunofluorescence staining of Iba-1 (green) and MPO (green) at 24 h after SAH. **b**, **e** Quantitative analyses of Iba-1- and MPO-positive cells in the basal cortex at 24 h after SAH. **P < 0.01 vs. sham, *##P* < 0.01 vs. SAH + vehicle. Error bars were represented as mean ± SD. *n* = 4 per group. **c**, **f** Representative western blot bands and quantitative analyses of Iba-1 and MPO protein levels in the left hemisphere at 24 h after SAH. **P < 0.01 vs. sham, ##*P* < 0.01 vs. SAH + vehicle. Error bars were represented as mean ± SD. *n* = 6 per group. *Bexa* bexarotene

The immunofluorescence staining of Iba-1- and MPO-positive cells and Western blot of Iba-1 and MPO protein levels were performed to access the microglia/macrophage activation and neutrophil infiltration in sham, SAH + vehicle, and SAH + Bexa groups at 24 h after SAH. There were significantly increased microglia activation and peripheral neutrophils infiltration at 24 h after SAH (Fig. 4). Immunofluorescence staining indicated that bexarotene treatment significantly decreased the number of activated Iba-1-positive microglia and the number of MPO-positive neutrophils in the basal cortex area of SAH rats (*P* < 0.05, Fig. 4a, b, d, e). Consistently, the Western blot results showed that the protein levels of Iba-1 and MPO in the left hemisphere were significantly decreased by bexarotene treatment when compared with vehicle-treated SAH rats at 24 h after SAH (*P* < 0.05, Fig. 4c, f).

### Bexarotene increased the RXR/PPARγ interaction at 24 h after SAH

**Fig. 5:**
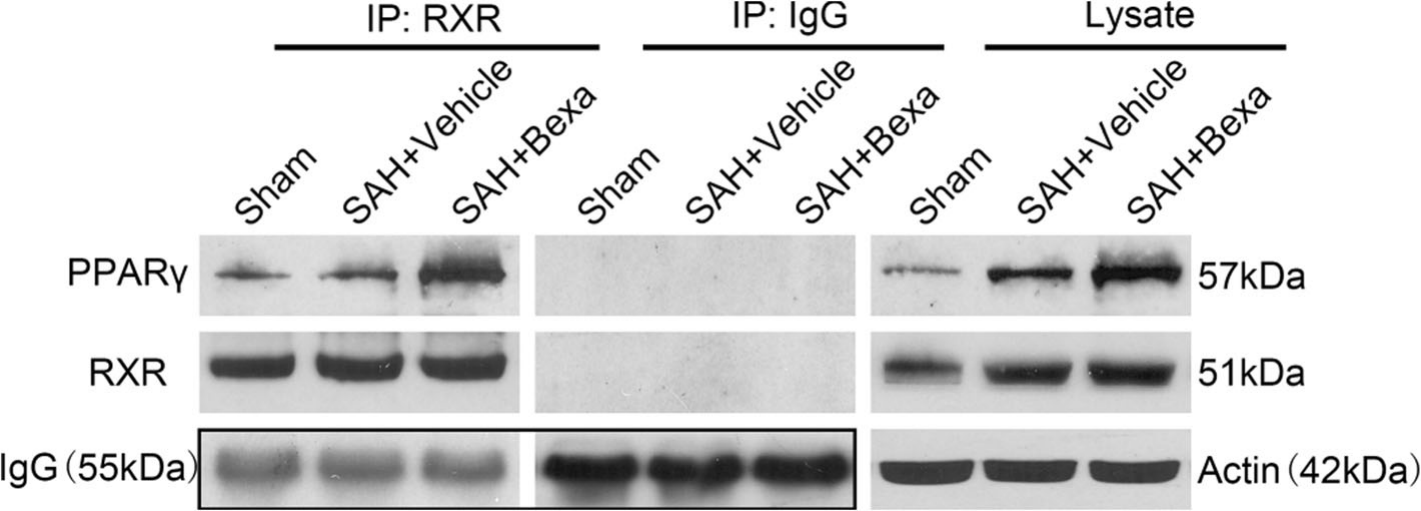
The effects of bexarotene on the RXR/ PPARγ interaction after SAH. IP assay of RXR/PPARγ interaction in left hemisphere at 24 h after SAH. *Bexa* bexarotene

Immunoprecipitation was applied to detect the RXR/PPARγ interaction by bexarotene administration at 24 h after SAH. The IP result showed that the RXR interact with PPARγ in sham and vehicle conditions; however, it was increased after bexarotene treatment (Fig. 5). These results suggested that the formation of RXR/PPARγ heterodimer were increased after bexarotene administration.

### Bexarotene treatment improved long-term neurobehavioral deficits after SAH

**Fig. 6:**
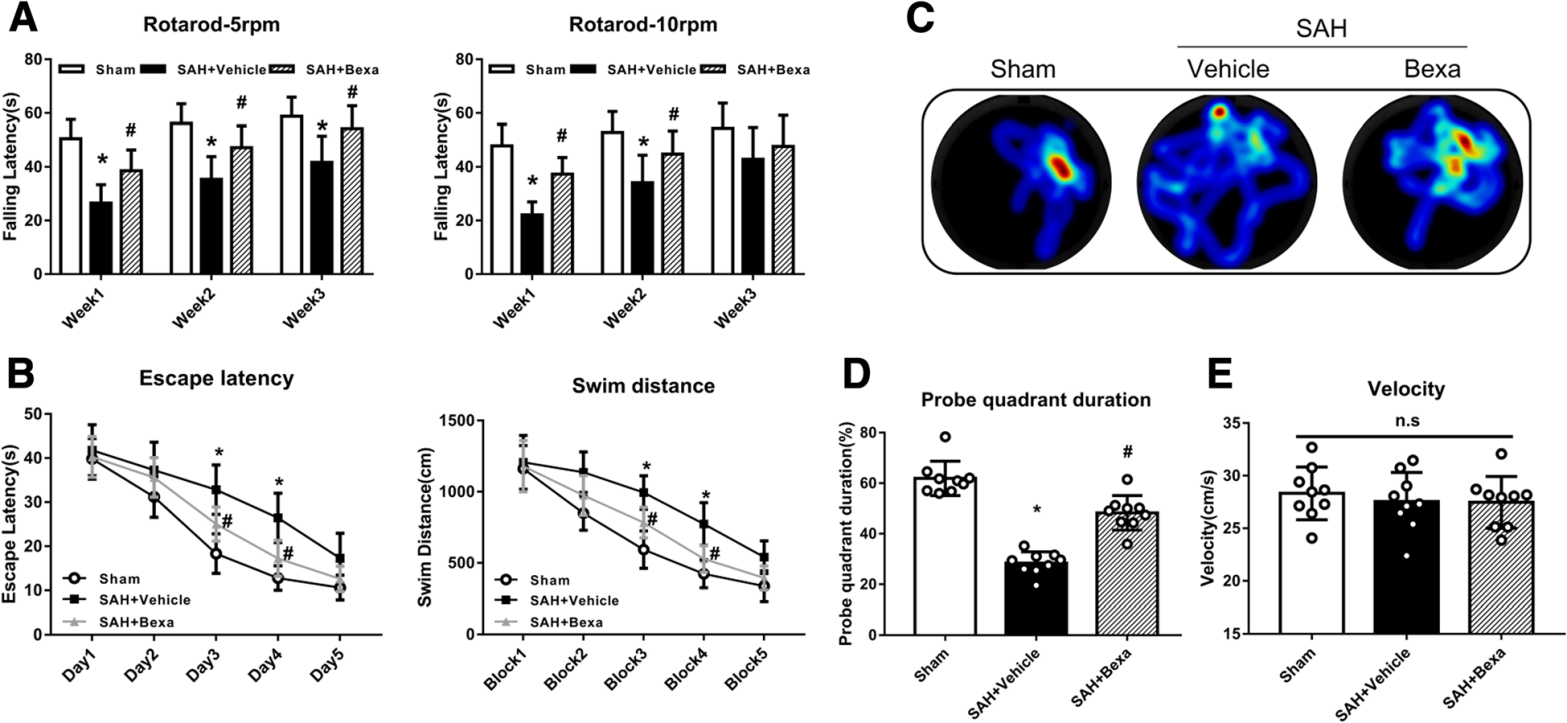
Bexarotene improved the long-term neurological deficit after SAH. **a** Rotarod test of 5 rpm and 10 rpm. **b** Escape latency and swim distance of water maze test. **c** Representative heat map in probe test showed bexarotene treated SAH spent more time in probe quadrant. **d** Probe quadrant duration. **e** velocity of probe trial. *P < 0.05, vs. sham group; #*P* < 0.05, vs. SAH + vehicle group. Data was represented as mean ± SD. *n* = 9 per group. *Bexa* bexarotene

Both the rotarod test and Morris water maze test were used to access the long-term neurological functions after SAH. In the rotarod test, the SAH + vehicle group had a significantly shorter falling latency at both 5 rpm and 10 rpm accelerating velocity tests when compared with the sham group in the 1 and 2 weeks after SAH. However, bexarotene treatment improved the rotarod performance of SAH rats at both 5 rpm and 10 rpm velocities compared to SAH + vehicle group (*P* < 0.05; Fig. 6a).

In the Morris water maze, the escape latency and swim distance for the rats to find the platform were significantly increased in the SAH + vehicle group when compared to the sham group (*P* < 0.05, Fig. 6b). However, a significant decrease in escape latency on days 3 to 4 and a significantly shorter swim distance on block 3 to 4 were observed in the SAH + Bexa group compared to the SAH + vehicle group (*P* < 0.05, Fig. 6b). In the probe quadrant trials, the percentage of time spent in the target quadrant was notably decreased in SAH + vehicle group compared with that in the sham group. However, bexarotene significantly increased the time spent in the target quadrant (*P* < 0.05; Fig. 6c, d). The swimming speed was not significantly different among all the groups (Fig. 6e).

### RXR antagonist UVI3003 reversed the anti-neuroinflammation effects of bexarotene and its regulation on PPARγ/SIRT6/p-FoxO3a protein levels at 24 h after SAH

**Fig. 7:**
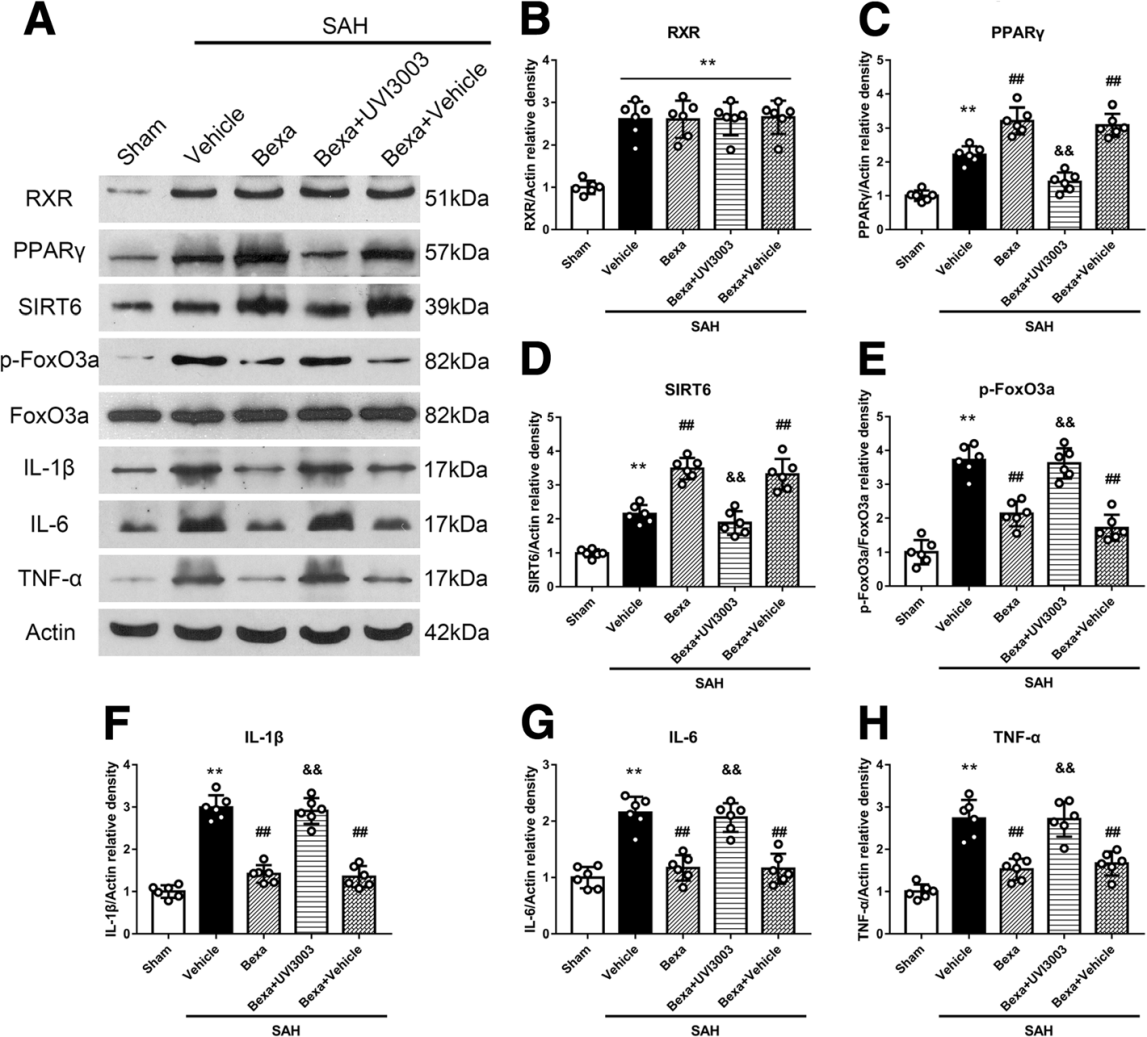
RXR selective inhibitor UVI3003 reversed the anti-neuroinflammation effects of bexarotene and its regulation on downstream signaling proteins of PPARγ/SIRT6/p-FoxO3a at 24 h after SAH. **a** Representative western blot bands. **b**–**h** Quantitative analyses of RXR, PPARγ, SIRT6, p-FoxO3a, IL-1β, IL-6, and TNF-α in the left hemisphere at 24 h after SAH. **P < 0.01 vs. sham, ##*P* < 0.01 vs. SAH + vehicle, and &&*P* < 0.01 vs. SAH + BEXA + vehicle. Error bars were represented as mean ± SD. *n* = 6 per group. *Bexa* bexarotene

To verify the RXR activation involved in the neuroprotective effects of bexarotene post-SAH, the specific RXR antagonist UVI3003 was injected via i.c.v to inhibit the RXR. Without changing RXR protein level, bexarotene further increased protein levels of PPARγ and SIRT6 but decreased p-FoxO3a and pro-inflammatory cytokines including IL-1β, IL-6, and TNF-a in SAH rats when compared with those in the SAH + vehicle group (*P* < 0.05; Fig. 7). UVI3003 reversed these effects of bexarotene after SAH (*P* < 0.05; Fig. 7).

### RXR knockdown reversed the anti-neuroinflammation effects of bexarotene and its regulation on PPARγ/SIRT6/p-FoxO3a protein levels at 24 h after SAH

**Fig. 8:**
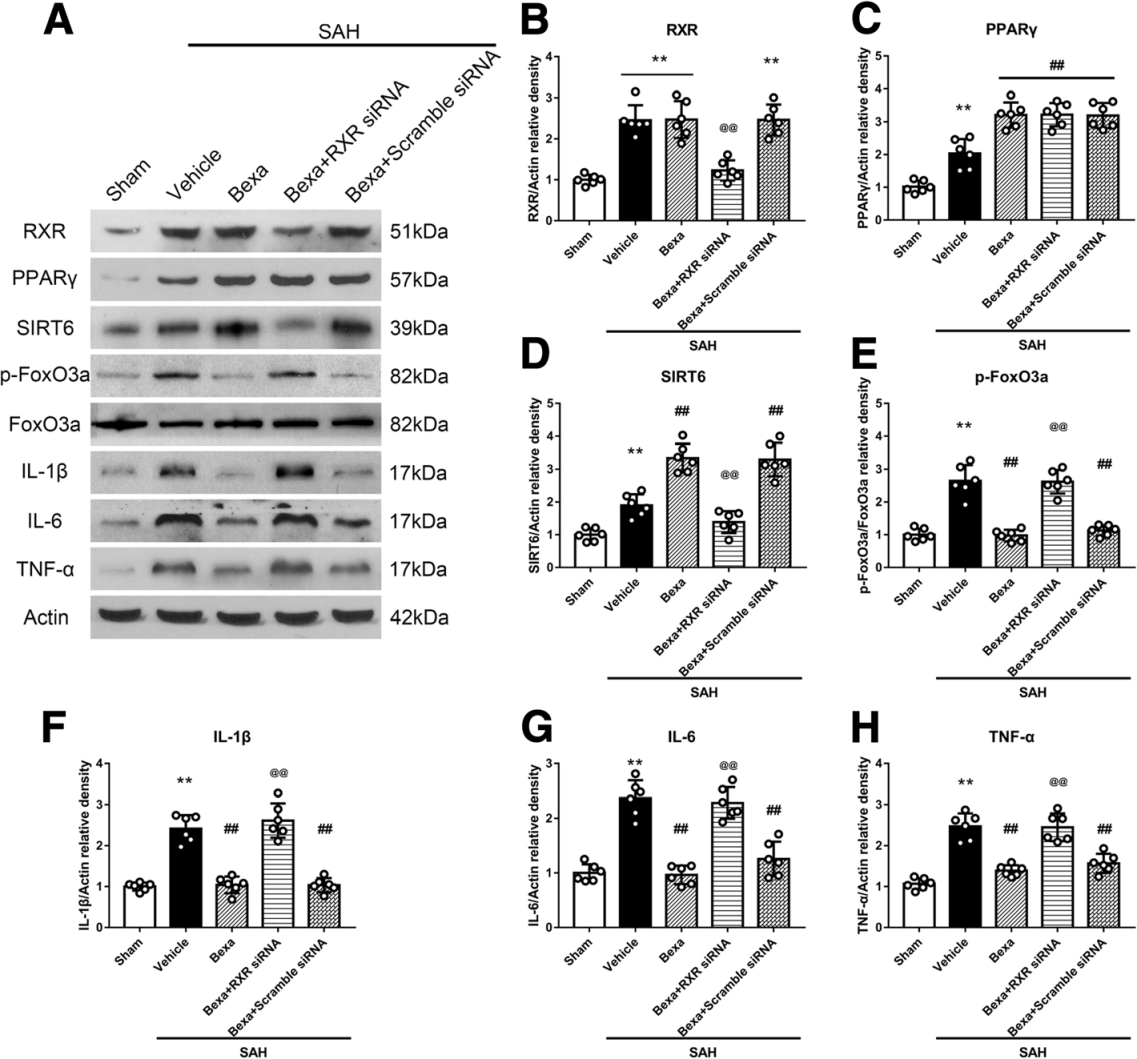
RXR knockdown reversed the anti-neuroinflammation effects of bexarotene at 24 h after SAH. **a** Representative Western blot bands. **b**–**h** Quantitative analyses of RXR, PPARγ, SIRT6, p-FoxO3a, IL-1β, IL-6, and TNF-α in the left hemisphere at 24 h after SAH. **P < 0.01 vs. sham, ##*P* < 0.01 vs. SAH + vehicle, and @@*P* < 0.01 vs. SAH + BEXA + scramble siRNA. Error bars were represented as mean ± SD. *n* = 6 per group. *Bexa* bexarotene

To further verify if the RXR mediated the neuroprotective effects of bexarotene post-SAH, rat RXR siRNA was injected by i.c.v to knockdown RXR in vivo. The expression of RXR was significantly decreased by RXR siRNA compared to scramble siRNA. Also, without affected PPARγ protein level, the downstream SIRT6 was decreased but increased p-FoxO3a and pro-inflammatory cytokines including IL-1β, IL-6, and TNF-a in RXR knockdown group when compared with scramble siRNA group (*P* < 0.05; Fig. 8). The results suggested that knockdown RXR reversed the neuroprotective effect of bexarotene after SAH.

### Selective SIRT6 inhibitor OSS128167 abolished the anti-neuroinflammation effects of bexarotene and its regulation on p-FoxO3a protein level at 24 h after SAH

**Fig. 9:**
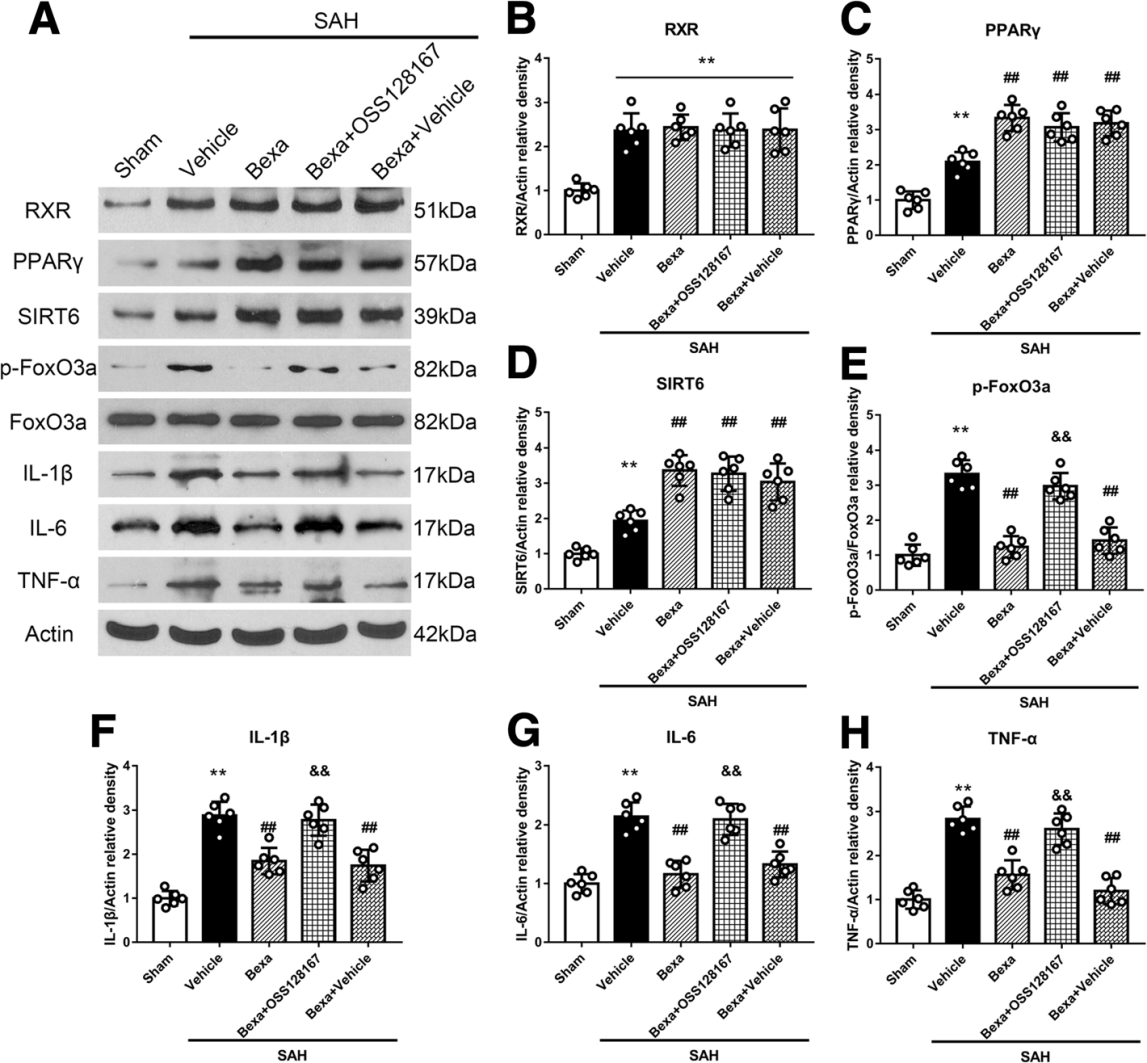
SIRT6 inhibitor OSS128167 reversed the anti-neuroinflammation effect of bexarotene and its regulation on downstream signaling protein of p-FoxO3a at 24 h after SAH. **a** Representative Western blot bands. **b**–**h** Quantitative analyses of RXR, PPARγ, SIRT6, p-FoxO3a, IL-1β, IL-6, and TNF-α in the left hemisphere at 24 h after SAH. **P < 0.01 vs. sham, ##*P* < 0.01 vs. SAH + vehicle, and &&*P* < 0.01 vs. SAH + Bexa + vehicle. Error bars were represented as mean ± SD. *n* = 6 per group. *Bexa* bexarotene

To verify if the SIRT6 was the downstream signaling of RXR activation by bexarotene, the selective SIRT6 inhibitor OSS128167 was injected via i.c.v to intervene the pathway of PPARγ/SIRT6/FoxO3a. The change of the proteins levels in the signaling pathway after bexarotene treatment was the same as Figs. 7 and 8. However, inhibiting SIRT6 by OSS128167 abolished the effects of bexarotene on p-FoxO3a and pro-inflammatory cytokines IL-1β, IL-6 and TNF-a (*P* < 0.05; Fig. 8a, e–h), but not the expression of PPARγ and SIRT6 (*P* > 0.05; Fig. 9a–c).

### Bexarotene attenuated SAH-induced IL-1β-positive cells and neutrophil infiltration which were abolished by either UVI3003 or OSS128167 at 24 h after SAH

**Fig. 10:**
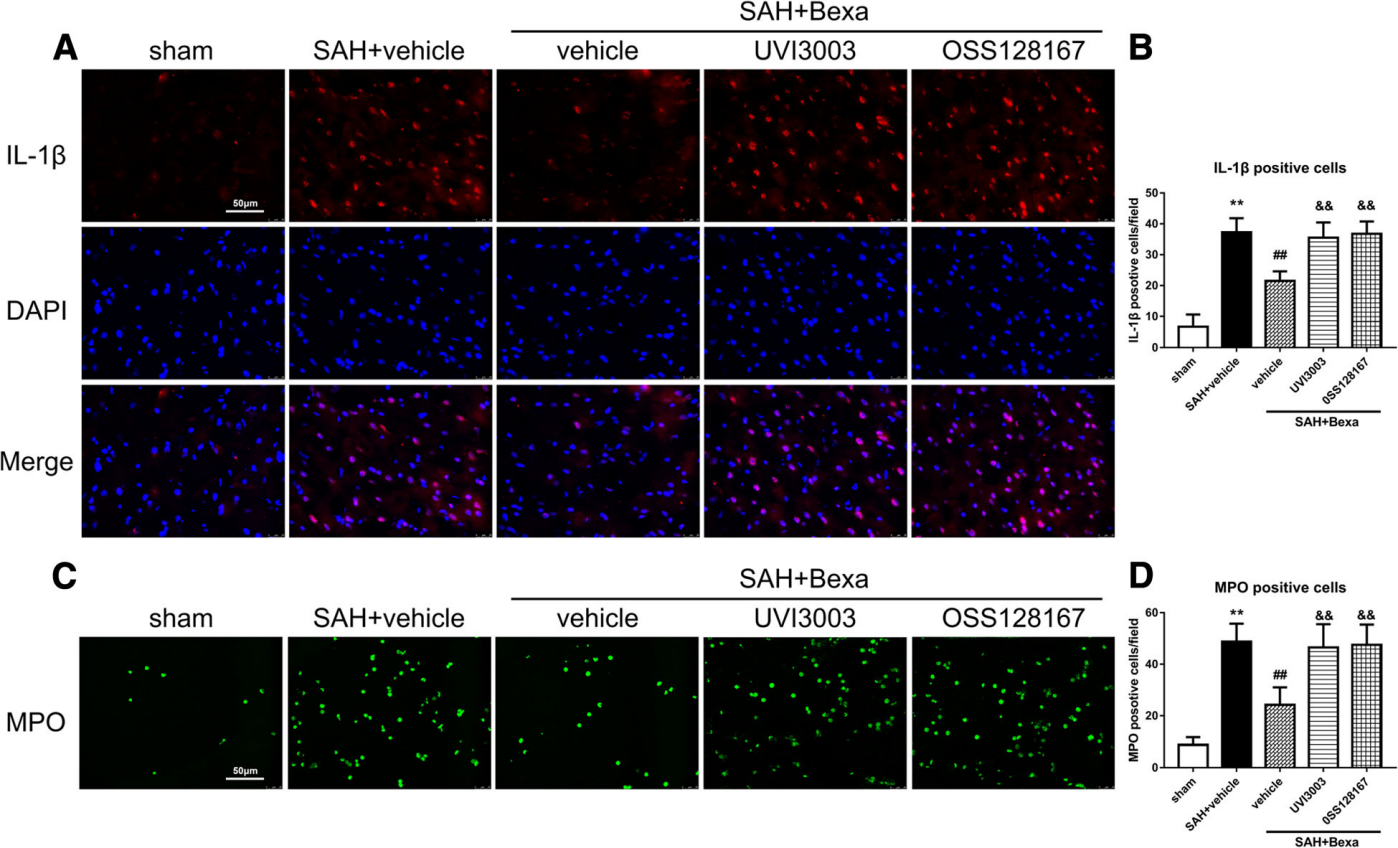
Bexarotene attenuated IL-1β-positive cells and MPO-positive cells infiltration at 24 h after SAH, which were abolished by UVI3003 or OSS128167. **a** Representative microphotography of IL-1β immunofluorescence staining in the left basal cortex. **b** Quantitative analyses of IL-1β-positive cells at 24 h after SAH. **c** Representative microphotography of MPO immunofluorescence staining in the left basal cortex. **d** Quantitative analyses of MPO-positive cells at 24 h after SAH. Scale bar, 50 μm. **P < 0.01 vs. sham, ##*P* < 0.01 vs. SAH + vehicle, and &&*P* < 0.01 vs. SAH + Bexa + vehicle. Error bars were represented as mean ± SD. *n* = 4 per group. *Bexa* bexarotene

Immunofluorescence staining of IL-1β and MPO were performed to confirm further that UVI3003 and OSS128167 abolished the anti-neuroinflammation effect of bexarotene after SAH. While the numbers of IL-1β- and MPO-positive cells were notably increased at 24 h after SAH, bexarotene treatment significantly inhibited these pro-inflammatory responses. The administration of either UVI3003 or OSS128167 significantly reversed the anti-neuroinflammation effects of bexarotene after SAH (*P* < 0.05, Fig. 10).

## Discussion

In the present study, we explored the neuroprotective effects of bexarotene and the potential underlying mechanisms after experimentally induced SAH in rats. Our results demonstrated that (1) brain RXR protein level was increased at an early stage and peaked at 24 h after SAH. RXR expressions were co-localized with neurons, astrocytes, and microglia. (2) Activation RXR by bexarotene improved short- and long-term neurobehavioral outcomes, ameliorated brain edema, as well as inhibited the microglial activation and neutrophil infiltration after SAH. (3) Bexarotene treatment significantly increased expressions of PPARγ and SIRT6 but decreased the FoxO3a phosphorylation and the expressions of pro-inflammatory cytokines. (4) RXR antagonist UVI3002, RXR knockdown, or SIRT6 inhibitor OSS12817 abolished the anti-neuroinflammatory effects of bexarotene and its effects on PPARγ/SIRT6/FoxO3a signaling pathway. Taken together, our results indicated that RXR activation with bexarotene might ameliorate neuroinflammation after SAH via at least partially the PPARγ/SIRT6/FoxO3a pathway.

Retinoid X receptor (RXR) is a nuclear factor widely expressed in the brain. Upon activation by binding to its ligands, RXR display a variety of biological functions [32]. Mounting of pieces of evidence suggested that RXR plays a vital role in the innate as well as in the adaptive immune response by regulating functions of monocytes/macrophages, dendritic cells, and T cells [6, 33]. In the present study, our results showed that the expression of RXR increased at the early stage of SAH, and co-localized with neurons, astrocytes, and microglia. The increased RXR expression may suggest its participation in the endogenous neuroprotection mechanisms after SAH. Cells uptake retinol from the bloodstream and convert it into retinoic acid. The 9-cis retinoic acid (9cRA) is considered to be the endogenous ligand that activates RXR to manifest the subsequent transcriptional effects [34, 35]. However, the efficacy of endogenous 9cRA in RXR activation is limited since it is usually under detectable concentration in the brain after stroke [36].

Bexarotene is a highly selective and blood-brain barrier permeable agonist of RXR, Additional file 2: Figure S3), which is initially approved by FDA for treating cutaneous T cell lymphoma [37]. Emerging studies demonstrated that bexarotene was neuroprotective by reducing inflammation, attenuating apoptosis, enhancing autophagy, and promoting myelin debris phagocytosis in the central nervous system (CNS) diseases such as Alzheimer’s disease, traumatic brain injury, and ischemic stroke [7, 16, 19, 38–41]. Consistent with the previous studies, our results showed that bexarotene treatment improved neurological deficit, reduced the brain edema, and attenuated the microglial activation and neutrophil infiltration at 24 h after SAH. Because inflammation causes blood-brain barrier disruption which exacerbates brain edema after SAH [8, 42], this also can be explained as bexarotene can further improve neurological outcome by ameliorating brain edema exacerbated by inflammation. Additionally, the hippocampal damage at an early stage after SAH could cause long-term cognitive and memory impairment [43]. In the present study, bexarotene single administration improved long-term neurobehavioral functions including the performance of rotarod test and Morris water maze spatial learning test. The long-term protection may be attributed to the attenuated EBI by RXR activation after SAH.

Heterodimers of RXR with PPARs are the most extensively studied nuclear receptors involved in immune responses that play crucial roles in apoptotic cell clearance, neutrophil homeostasis, immune cell proliferation, T cell differentiation, and inflammatory gene repression [44]. The RXR/PPARγ heterodimer activation has been shown to be neuroprotective in CNS diseases [7, 45, 46]. Recent studies indicated that the PPARγ activation showed a protective effect by increasing SIRT6 expression in animal models of hepatic steatosis and Huntington’s disease (HD) [13, 14]. Consistently, in the present study, our results demonstrated that administration of bexarotene significantly increased the RXR/PPARγ interaction, and upregulated the expression of SIRT6, while downregulating the pro-inflammatory cytokines including IL-1β, IL-6, and TNF-α at 24 h after SAH. UVI3003, a selective RXR antagonist [32, 47, 48], could ultimately prevent the beneficial effects of RXR activation in retinal degenerations [49]. Similarly, when we inhibited RXR using UVI3003 or knockdown RXR before SAH induction, the effects of bexarotene treatment on PPARγ/SIRT6 upregulation and neuroinflammation suppression as well as neurological deficit improvement were abolished at 24 h after SAH.

Both in vitro and in vivo studies demonstrated that SIRT6 produced beneficial effects in cerebrovascular diseases models [50–52]. SIRT6 was essential for the sodium sulfide-mediated cytoprotective effect in brain endothelial cells subjected to ischemia/reperfusion (I/R) injury [51]. SIRT6 protected the brain from cerebral I/R injury [50] and contributed to neurogenesis after cerebral ischemia [52]. Moreover, SIRT6 protected cardiomyocytes against I/R injury by a FoxO3a-dependent mechanism, in which SIRT6 decreased cellular oxidative stress by inhibiting FoxO3a phosphorylation and subsequently upregulating the antioxidant-encoding gene expression [15]. Consistently, we found that bexarotene treatment significantly increased SIRT6 expression but decrease the expression of p-FoxO3a and pro-inflammatory cytokines. The application of OSS128167, a selective SIRT6 inhibitor, reversed the neurological improvement and anti-inflammatory effects of bexarotene and its down-regulation on p-FoxO3a expression after SAH. Mechanistically, the RXR inhibitor, RXR knockdown, and SIRT6 inhibitor intervene the pathway, reversed the neuroscore improvement (Additional file 3: Figure S2) and the anti-neuroinflammatory effect marked by pro-inflammatory cytokines after SAH treated by bexarotene. Taken together, our results suggested that PPARγ/SIRT6/FoxO3a signaling pathway may underline the anti-neuroinflammation and neurological improvement of bexarotene mediated by RXR after SAH.

There were some limitations to the present study. First, we focused on the anti-inflammatory effects of bexarotene after SAH, but we cannot exclude the possibility that bexarotene may also exert other protective effects, such as preservation of BBB integrity, modulation of autophagy, and regulation neurogenesis [16, 39, 53]. Second, bexarotene administration was only applied at a single time point (1 h after SAH). Thus the optimal therapeutic window of bexarotene treatment for SAH was not evaluated in the present study. Third, a previous study reported that activation of PPARγ attenuated NF-κB-mediated inflammation [8]. Therefore, there may be other downstream signaling pathways underlying the anti-inflammation effects of bexarotene. Future studies are required to fully elucidate the other neuroprotective functions and potential mechanisms associated with bexarotene in the setting of experimental SAH.

## Conclusions

Our findings demonstrated that the activation of RXR with bexarotene improved the short- and long-term neurological functions, and attenuated neuroinflammation after SAH in rats. The protective effects of bexarotene were at least in part through PPARγ/SIRT6/FoxO3a signaling pathway. Therefore, bexarotene may provide a promising therapeutic strategy for the management of SAH patients.

## Additional files

Additional file 1: **Figure S1.** A. Numbers of animals used, mortality and exclusion in each group. B. Representative brain images in sham and SAH groups. Blood clots were mainly present around the Circle of Willis at 24 h after SAH. C. SAH grade scores of all SAH groups at 24 h after SAH. Bexa, bexarotene; UVI3003, a specific RXR antagonist; OSS128167, a selective SIRT6 inhibitor; Scr siRNA, Scramble siRNA; SAH, subarachnoid hemorrhage. (TIF 7668 kb)

Additional file 2: **Figure S3.** Detection of bexarotene in the brain after intraperitoneal administration. A. Mass spectra of bexarotene detected with full scan mass spectrometry (MS). B. MS/MS spectra of precursor ion at *m/z* 349 from bexarotene standard. C. MS/MS spectra of precursor ion at *m/z* 349 from the brain of dosed rats. (TIF 3101 kb)

Additional file 3: **Figure S2.** Bexarotene improves neurological deficit at 24 h after SAH, however the neuroprotective effect can be reversed by UVI3003, RXR knockdown and OSS128167 administration. A. Modified Garcia score. B. Beam balance test. **p < 0.01 vs. sham, ##*p* < 0.01 vs. SAH + vehicle, and &&*p* < 0.01 vs. SAH + Bexa + vehicle. @@*p* < 0.01 vs. SAH + Bexa + Scr siRNA. Error bars were represented as mean ± SD. *n* = 6 per group. Bexa, bexarotene; Scr siRNA, scramble siRNA. (TIF 1731 kb)

## Abbreviations

9cRA: 9-cis retinoic acid
AD: Alzheimer’s disease
ANOVA: Analysis of variance
Bexa: Bexarotene
CNS: Central nervous system
DMSO: Dimethyl sulfoxide
EBI: Early brain injury
FDA: Food and Drug Administration
FoxO3a: Forkhead box O3a
GFAP: Glial fibrillary acidic protein
HD: Huntington’s disease
i.c.v: Intracerebroventricular
i.p: Intraperitoneal
I/R: Ischemia/reperfusion
Iba-1: Ionized calcium binding adaptor molecule 1
MPO: Myeloperoxidase
NeuN: Neuronal nuclear
p-FoxO3a: Phosphate forkhead box O3a
PPARγ: Peroxisome proliferator-activated receptor gamma
RXR: Retinoid X receptor
SAH: Subarachnoid hemorrhage
SD: Standard deviation
SIRT6: Sirtuin 6
