# Supplementary figures and images for: Activation of retinoid X receptor by bexarotene attenuates neuroinflammation via PPARγ/SIRT6/FoxO3a pathway after subarachnoid hemorrhage in rats
Source: J Neuroinflammation. 2019 Feb 21;16:47. doi: 10.1186/s12974-019-1432-5 (PMC6385420; doi:10.1186/s12974-019-1432-5)

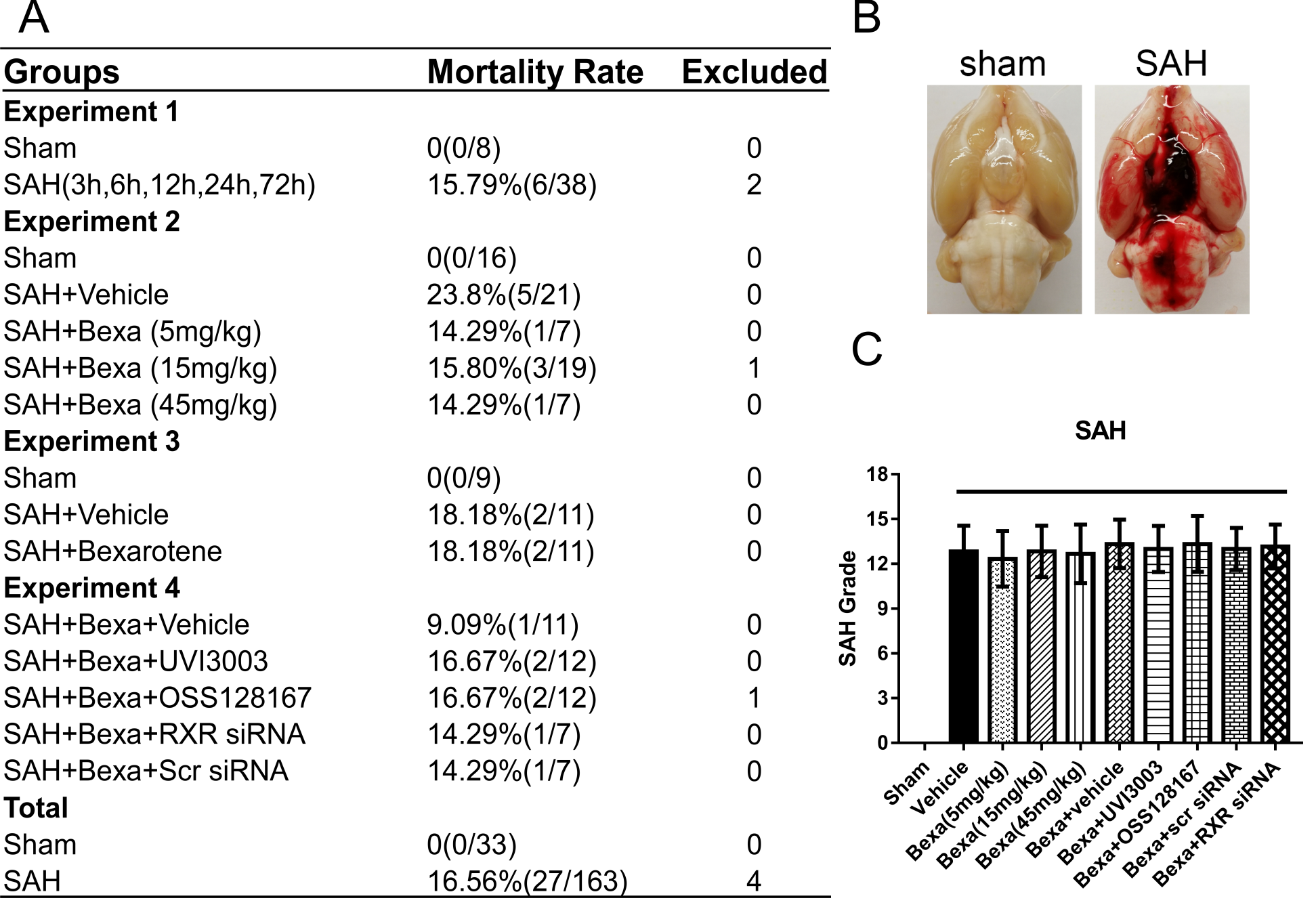

Supplement: Supplementary file 1 — Figure S1. A. Numbers of animals used, mortality and exclusion in each group. B. Representative brain images in sham and SAH groups. Blood clots were mainly present around the Circle of Willis at 24 h after SAH. C. SAH grade scores of all SAH groups at 24 h after SAH. Bexa, bexarotene; UVI3003, a specific RXR antagonist; OSS128167, a selective SIRT6 inhibitor; Scr siRNA, Scramble siRNA; SAH, subarachnoid hemorrhage. (TIF 7668 kb) [file 12974_2019_1432_MOESM1_ESM.tif]

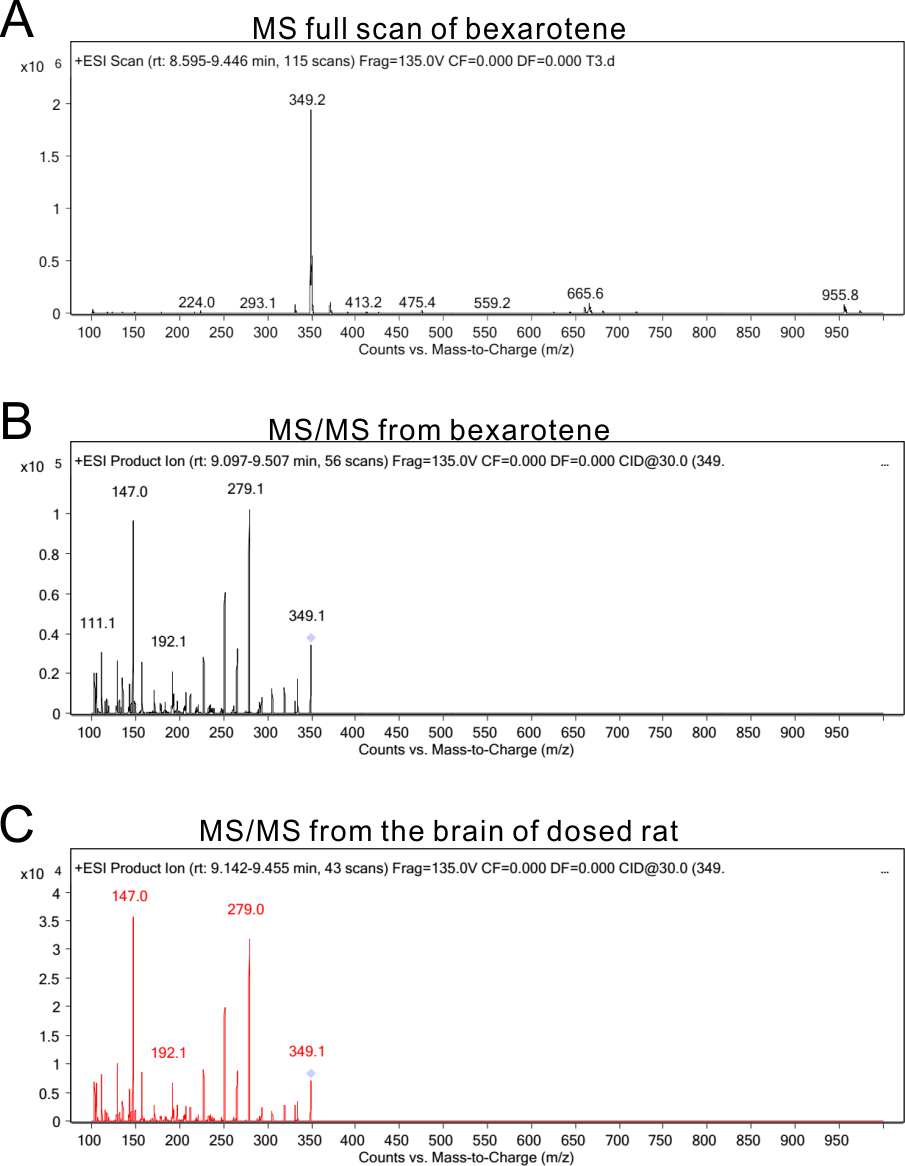

Supplement: Supplementary file 2 — Figure S3. Detection of bexarotene in the brain after intraperitoneal administration. A. Mass spectra of bexarotene detected with full scan mass spectrometry (MS). B. MS/MS spectra of precursor ion at m/z 349 from bexarotene standard. C. MS/MS spectra of precursor ion at m/z 349 from the brain of dosed rats. (TIF 3101 kb) [file 12974_2019_1432_MOESM2_ESM.tif]

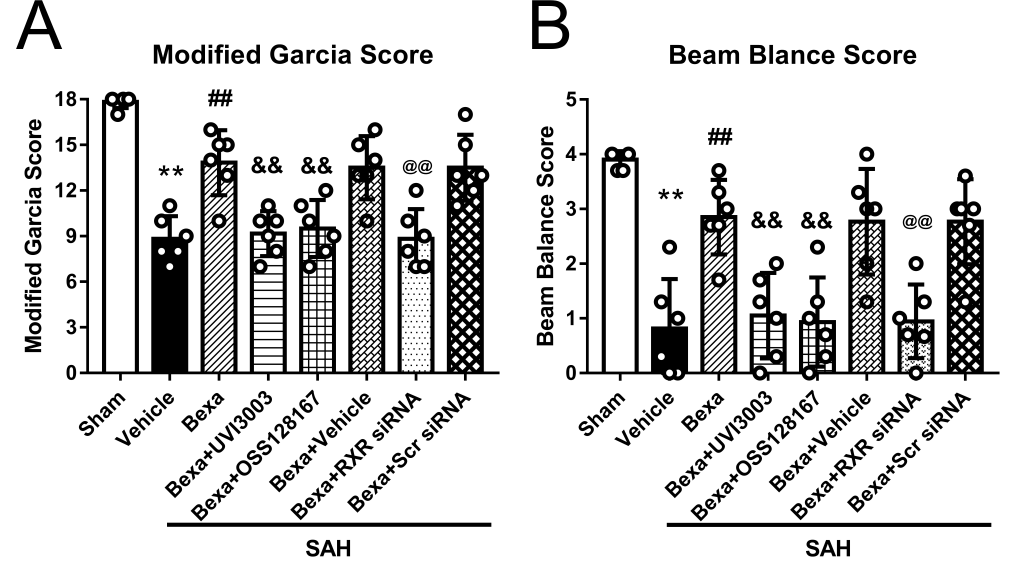

Supplement: Supplementary file 3 — Figure S2. Bexarotene improves neurological deficit at 24 h after SAH, however the neuroprotective effect can be reversed by UVI3003, RXR knockdown and OSS128167 administration. A. Modified Garcia score. B. Beam balance test. **p < 0.01 vs. sham, ##p < 0.01 vs. SAH + vehicle, and &&p < 0.01 vs. SAH + Bexa + vehicle. @@p < 0.01 vs. SAH + Bexa + Scr siRNA. Error bars were represented as mean ± SD. n = 6 per group. Bexa, bexarotene; Scr siRNA, scramble siRNA. (TIF 1731 kb) [file 12974_2019_1432_MOESM3_ESM.tif]
